# Recruitment methods in Alzheimer's disease research: general practice versus population based screening by mail

**DOI:** 10.1186/1471-2288-10-35

**Published:** 2010-04-29

**Authors:** Fred Andersen, Torgeir A Engstad, Bjørn Straume, Matti Viitanen, Dag S Halvorsen, Samuel Hykkerud, Kjell Sjøbrend

**Affiliations:** 1 Department of Community Medicine, University of Tromsø, N-9037 Tromsø, Norway; 2 Department of Geriatrics, University Hospital in Northern Norway, N-9038 Tromsø, Norway; 3 Department of Geriatrics, Karolinska Institutet, Karolinska University Hospital, Stockholm, Sweden; 4 University of Turku, Turku, Finland; 5 Vefsn Health Centre, N-8656 Mosjøen, Norway

## Abstract

**Background:**

In Alzheimer's disease (AD) research patients are usually recruited from clinical practice, memory clinics or nursing homes. Lack of standardised inclusion and diagnostic criteria is a major concern in current AD studies. The aim of the study was to explore whether patient characteristics differ between study samples recruited from general practice and from a population based screening by mail within the same geographic areas in rural Northern Norway.

**Methods:**

An interventional study in nine municipalities with 70000 inhabitants was designed. Patients were recruited from general practice or by population based screening of cognitive function by mail. We sent a questionnaire to 11807 individuals ≥ 65 years of age of whom 3767 responded. Among these, 438 individuals whose answers raised a suspicion of cognitive impairment were invited to an extended cognitive and clinical examination. Descriptive statistics, chi-square, independent sample t-test and analyses of covariance adjusted for possible confounders were used.

**Results:**

The final study samples included 100 patients recruited by screening and 87 from general practice. Screening through mail recruited younger and more self-reliant male patients with a higher MMSE sum score, whereas older women with more severe cognitive impairment were recruited from general practice. Adjustment for age did not alter the statistically significant differences of cognitive function, self-reliance and gender distribution between patients recruited by screening and from general practice.

**Conclusions:**

Different recruitment procedures of individuals with cognitive impairment provided study samples with different demographic characteristics. Initial cognitive screening by mail, preceding extended cognitive testing and clinical examination may be a suitable recruitment strategy in studies of early stage AD.

**Clinical Registration:**

ClinicalTrial.gov Identifier: NCT00443014

## Background

Alzheimer's disease (AD) constitutes 65 - 70% of all dementia subtypes. The prevalence is 10% for individuals 65 years and older, encompassing 90% of all AD. The incidence increases from <1% among people 65-69 years of age to nearly 9% above 85, making AD a major cause of disability in the elderly [1,2].

Due to the increasing lifespan and the decreasing ratio of working to retired populations, the social and economic burden of neurodegenerative diseases are growing [3] and may be threatening future welfare and health care. As a consequence, the European Science and Research Commission has declared that prevention, early identification, and postponement of AD onset should have high priority. In order to remove or reduce modifiable AD risk factors, increasing attention to cognitive impairment is needed within the medical communities, including more reliable early AD screening tools.

Characteristics of AD study participants depend on study design, inclusion and diagnostic criteria, recruitment method and age distribution [4,5]. In clinical trials AD patients are usually recruited from memory clinics, hospitals and nursing homes, which makes the studies prone to selection bias [6]. The heterogeneity of diagnostic criteria and diagnostic tools reinforce these methodological challenges [7-10]. As a consequence few validated screening questionnaires are available and study comparisons may be hampered due to lack of standardization [11-13]. In a recent population based study Palmer et al showed that mild cognitive impairment criteria failed to identify individuals with global cognitive deficits at high risk of AD progression [14].

The impact of different recruitment methods on sample characteristics is insufficiently examined [15], whereas studies comparing sample characteristics of individuals recruited by different methods from the same population are lacking.

The aim of this paper is to compare clinical and demographic characteristics in AD individuals recruited from general practice or by population based screening questionnaires in the same geographical area.

## Methods

### Participants

The Dementia Study in Rural Northern Norway planned to recruit 200 patients with recently diagnosed AD in primary health care in nine rural municipalities, from January 2006 to December 2007. AD patients were recruited from general practice (n = 87) and through population based screening (n = 100) based on the same diagnostic criteria. Both groups underwent similar cognitive, physical and laboratory examinations. Inclusion criteria were individuals aged ≥ 65 years with a MMSE sum score ≥ 10 and ≤ 30 points. Exclusion criteria were delirium and behavioral disturbances interfering with cognitive and clinical testing, reluctance to participate, and inability to understand the purpose of the study, or relatives/caregivers disapproving participation.

### Recruitment by population based screening

Due to a low inclusion rate by general practitioners (GPs) during the first year, the recruitment method was extended to comprise a population based screening of cognitive impairment. An invitation letter enclosing a questionnaire modified from the Cambridge Examination for Mental Disorders of the Elderly and Strawbridge et al [11,12] was sent to 11807 individuals ≥ 65 years of age in the participating municipalities. The first question was on participation (*Do you want to participate in the Dementia study?)*. Four questions covered memory impairment (*Have your memory deteriorated?*), visuo-spatial skills (*Do you forget where objects were left?*), speech difficulties (*Do you have difficulties to find the appropriate words?*) and activities of daily living (*Do you have difficulties in managing daily activities, which earlier represented no problem?*). An algorithm was designed to identify individuals at increased risk of having cognitive impairment (Appendix 1). Based on this, 438 individuals were invited to an extended cognitive and clinical examination. A physician with background in geriatric medicine examined the invited individuals, and supervised and completed the interdisciplinary diagnostic procedures. Among the respondents without self reported cognitive impairment, a randomly selected reference group, undergoing cognitive testing, was established.

### Examination of individuals with memory problems

Prior to study start, GPs and co-workers were trained to identify and diagnose AD based on the Norwegian guidelines [16]. A semi-structured interview of the participants focused on the onset and the course of cognitive impairment emphasising memory and visuo-spatial disturbances, word-finding difficulties, and changes in executive functions including activities of daily living (ADL). A family member or a caregiver was encouraged to add medical data according to the Informant Questionnaire-Cognitive Decline in the Elderly (IQ-CODE) [17]. Cognitive function was tested with The Mini Mental Status Examination (MMSE) [18] and Clock drawing test [19], and depression was examined with Montgomery and Aasberg Depression Rating Scale (MADRS) [20]. Neurological examination, blood tests and cerebral computed tomography (CT) were performed to exclude other causes of dementia but AD.

### AD diagnosis

The diagnosis of dementia was set by GPs and discussed with at least one specialist in geriatric medicine according to the ICD-10 criteria [21], AD according to the Statistical Manual of Mental Disorders fourth edition (DSM-IV-TR) and probable AD according to National Institute of Neurological Disorders and Stroke-Alzheimer Disease and Related Disorders' (NINCDS-ADRDA) criteria [22]. Disagreement or uncertainty about the diagnostic subtypes regarding 12 patients was solved by consulting a third specialist in geriatric medicine (MV).

### Approvals

The present study is registered as an International Standard Randomised Controlled Trial within ClinicalTrials.gov and approved by the following bodies; The Regional Committee for Medical Research Ethics in Northern Norway, The Privacy Ombudsman for Research, The Directory of Health and Social Welfare and The Norwegian Medicine Agency including the EudraCT database (no 2004-002613-37). The Norwegian Medicine Agency concluded that the study was conducted according to the principles of Good Clinical Practice. Each participant gave a written informed consent co-signed by a spouse, a close relative or a guardian. The national authorities listed above have approved the consent formula.

This manuscript intends to comply with The CONSORT statements and The Uniform Requirements for Manuscripts Submitted to Biomedical Journals.

### Statistics

Descriptive statistics, chi-square, independent sample t-test, analyses of covariance adjusted for possible confounders and multivariable analysis were used with a two-sided significance level of 5%. SPSS version 15 was applied for both data management and analysis.

## Results

At the end of the inclusion period 87 patients were recruited from GPs and 100 from population based postal screening.

**Figure 1:**
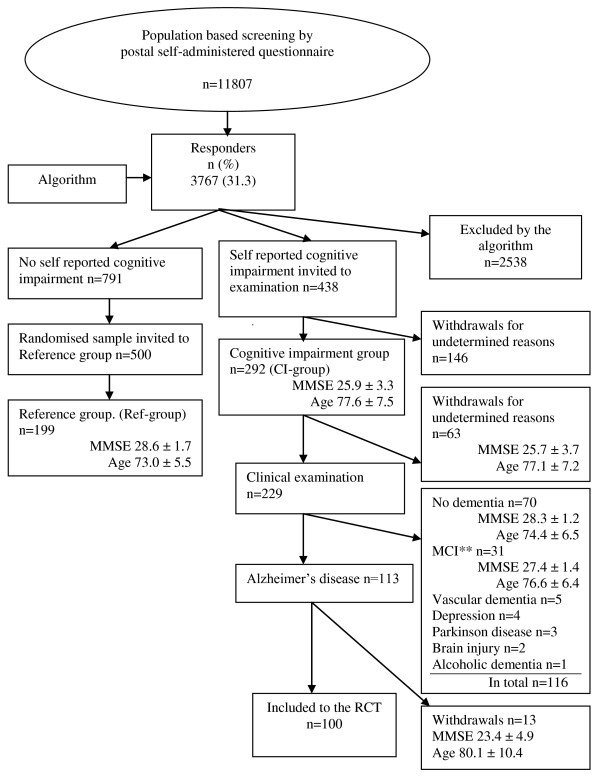
**Flowchart of population based recruitment to the study by postal screening of cognitive function followed by clinical examination and testing**. *Selection according to a predefined algorithm, Appendix 1. ** Mild cognitive impairment.

Figure 1 describes in detail the outcome of the screening method. 3767 (31.3%) individuals responded to the questionnaire, of which 438 persons met the criteria of self reported cognitive impairment. The cognitive impairment (CI) group consisted of 292 individuals, but only 229 individuals underwent cognitive and clinical examinations due to withdrawals.

Seventy of these had no dementia, 31 had mild cognitive impairment [23] and 15 had cognitive impairment not due to AD. One hundred and thirteen individuals had probable AD constituting 49% of the clinically examined group (n = 229). Thirteen patients with probable AD withdrew before inclusion. Of those examined, but not included, 53% were women.

Among 791 individuals who answered "Yes" to question one (participation) and "No" to the remaining five screening questions, 500 individuals were randomly selected as references for the cases. The final reference group (Ref-group) comprised 199 individuals who underwent cognitive testing. A highly significant difference (p < .001, age adjusted) was found for MMSE sum score between individuals with and without self-reported cognitive impairment (CI-group versus Ref-group, Figure 1).

A comparison between the two recruitment methods (screening versus general practice) (Table 1) revealed that AD patients recruited by screening more often were male (p < .001), younger (p = .006), needed less community support (p < .001), and had a significantly higher MMSE sum score (p < .001) as compared to those recruited in general practice, also when adjusting for education and co-morbidity (p < .001). In a multivariable model, the estimates remained unchanged. Overall, men were younger than women (p = .001). Compared to men, women more frequently lived alone (p < .001, age adjusted) and more frequently needed support from community care (p = .04, age adjusted).

**Table 1:** Demographic characteristics according to gender and recruitment method.

	Total	Routine Practice	Screening	p-value
Age Years (n)				
All	80.7 ± 7.0 (187)	82.3 ± 6.1 (87)	79.5 ± 7.5 (100)	= .006
Women	82.0 ± 6.5 (113)	83.0 ± 5.5 (67)	81.1 ± 7.5 (46)	= .13
Men	78.8 ± 7.2 (74)	80.2 ± 7.5 (20)	78.2 ± 7.1 (54)	= .29
p-value	= .001	= .08	= .06	
				
Gender, women n (%)				
	113 (60)	67 (77)	46 (46)	< .001
				
MMSE score				
All	23.0 ± 3.9	21.3 ± 4.2	24.4 ± 2.9	< .001*
Women	22.5 ± 3.9	21.2 ± 4.1	24.4 ± 2.7	< .001
Men	23.8 ± 3.7	21.8 ± 4.6	24.5 ± 3.0	= .004
p-value	= .03†	= .60	= .80	
				
Marital status (living couple) n (%)				
All	81 (43)	28 (32)	53 (53)	= .004
Women	29 (26)	15 (22)	14 (30)	= .34
Men	70 (52)	13 (65)	39 (72)	= .40
p-value	< .001§	= .001	< .001	
				
Own house/compartment n (%)				
All	141 (75)	54 (62)	87 (87)	< .001
Women	77 (68)	41 (61)	36 (78)	= .06
Men	64 (86)	13 (65)	51 (94)	= .001
p-value	= .004||	= .76	= .04	
				
Support from community nurses n (%)				
All	85 (47)	57 (66)	28 (28)	< .001
Women	62 (55)	45 (67)	17 (37)	= .002
Men	23 (32)	12 (60)	11 (21)	= .001
p-value	= .002.¶	= .55	= .08	

The Northern Norwegian Dementia Study Group 2008.  *Adjusted for age, education, gender and co-morbidity p < .001. †Age-adjusted p = .17. §Age-adjusted p < .001. ||Age-adjusted p = .10. ¶Age-adjusted p = .04

## Discussion

The main finding of this comparative study was that AD patient characteristics differed according to recruitment method. Younger and more often male patients with a higher MMSE score were recruited by mail as compared to those recruited in general practice. The estimate of baseline characteristics remained significantly different when adjusting for education, coronary heart disease, hypertension, stroke and diabetes. However, the overall gender difference in MMSE score turned non-significant when adjusting for age.

54% of those recruited by population based screening were men in contrast to only 23% from general practice. This is in accordance with the findings of Fitzpatrick et al who reported that men younger than 85 years were more willing to attend clinical trials dealing with cognitive function. According to Norwegian Statistics 2008, the male proportion of the general population above 70 and 80 years were 42% and 35%, respectively [24].

Men recruited by screening were more frequently living in a relationship in their own home without community support as compared to men recruited from general practice. In contrast, female participants were older, less self-reliant and more likely to live alone. All these factors are probably highly inter-correlated, partly as a result of different life expectancies for men and women. Living alone may promote inactivity, isolation and stimulus deprivation, all contributing to dementia progression [25].

Our study confirms that different recruitment methods in AD research provide samples with different baseline characteristics. Similar findings have been reported by Izal et al who emphasises that this could influence the results significantly [5]. A possible explanation is that a mailed questionnaire may alert those with a concern of own memory problems or early stage dementia whereas routine practice usually diagnoses patients with more obvious cognitive impairment and loss of compliance and self-care. According to the algorithm of the screening program 438 persons were invited to a subsequent cognitive and clinical examination, of which 113 (26%) were diagnosed with probable AD. In a large population based screening program conducted by Crews et al, 44.3% of the participants were recommended a follow-up. Of these, 24% were referred for objective memory impairment. A number of the patients reported that their GPs had never adequately assessed their memory complaints [26].

As for the present screening program, we found that a selection based on the answers to the postal questionnaire resulted in samples with highly significant differences in cognitive abilities (p < .001 age adjusted). Among 229 individuals undergoing clinical examination 70 (30%) had no dementia but had a family member or close relative with such a diagnosis, which probably contributed to a higher level of concern regarding cognitive symptoms. This group (n = 70) had similar MMSE sum score as the Ref-group.

Study limitations include the relative low number of participants in both groups. If screening questions are imprecise, information bias may threaten the internal validity of a study like this. We used questions based on the Cambridge Cognitive Examination, a widely accepted and reliable screening tool [11]. Our results indicate that the questions are capable of identifying individuals with MMSE scores corresponding to early AD. In our opinion, it is likely that the results are valid for western populations with similar demographics and co-morbidities.

It is known that GPs hesitate to diagnose mild cognitive impairment or early stage of dementia [27]. Mild to moderate cognitive impairment in the elderly, including early stage AD, seems to be disregarded by both relatives and health professionals, even though this stage of cognitive impairment has the best response to intervention. In this study, only one of five GPs in the nine municipalities joined the pre-study educational program aimed to improve AD diagnostics. Our findings are in accordance with those of Vernooij-Dassen et al who reported that GPs tend to postpone a comprehensive examination of patients who complain of memory problems [28]. Lack of therapeutic and diagnostic skills may contribute to this attitude [27]. Carters et al described such insufficient examination as a consequence of time constraints [29], whereas Wilcock et al reported that a number of GPs considered studies aiming to diagnose and manage AD irrelevant to their practice [30].

According to Norwegian recommendations, early stage dementia and MCI should be diagnosed in memory clinics and not in primary health care [31]. However, due to shortage of memory clinics, this strategy will not benefit the majority of elderly with early stage dementia. Under these circumstances GPs have to be supported and trained to detect and diagnose cognitive impairment.

## Conclusions

Few AD studies have compared patient characteristics of different recruitment methods. In our study patients recruited by screening were younger, more frequently men and had a higher MMSE sum score as compared to those recruited from general practice. Screening of cognitive function by questionnaires enables recruitment of early stage AD, whereas general practice recruits patients with more advanced disease. Future dementia studies on prevention and treatment should take recruitment methods into consideration, in particular when focusing on early stage AD. According to our experience, the applied screening questionnaire, preceding a clinical examination, would be a suitable strategy to identify and diagnose AD in primary health care.

## Competing interests

The authors declare that they have no competing interests.

## Authors' contributions

FA has initiated, coordinated and conducted this study in close co-operation with the scientific advisory board at The University of Tromsø. He has examined and diagnosed patients recruited both in general practice and by postal cognitive screening. He is also responsible for analysing baseline data. TE has been the main supervisor and member of the scientific advisory board, participating in all stages of this study; - planning, lecturing, collecting data, discussing results and writing. BS participated in the planning of the study, supervising implementation and analysis and has revised the manuscript. He is a member of the scientific advisory board. MV is a member of the scientific advisory board and has participated in diagnosing 12 patients, and in revising this manuscript. DH has participated in the data analysis, and has contributed in drafting and writing of the paper. SH has participated in the development of the data-recording file and supervised the training of the test technicians. He also has examined and diagnosed a number of patients in the study. KS has examined and diagnosed a number of patients recruited both in general practice and by postal cognitive screening.

All authors have approved the final version of the paper.

## Pre-publication history

The pre-publication history for this paper can be accessed here:

http://www.biomedcentral.com/1471-2288/10/35/prepub

## Appendix 1

### Postal Questionnaire

1. Do you want to participate in the Dementia study?

2. Have your memory deteriorated?

3. Do you forget where objects were left?

4. Do you have difficulties to find the appropriate words?

5. Do you have difficulties in managing daily activities, which earlier represented no problem?

6. Have you been examined for memory impairment earlier?

### Algorithm

Patients invited to clinical examination

1. "Yes" on question 1, 2, 3, 4, 5. "No" on question 6

2. "Yes" on question 1, 2, 5 and 3 or 4. "No" on question 6

Persons randomised and invited to participate in reference group

3. "Yes" on question 1 (participation) "No" on question 2, 3, 4, 5 and 6
